# Uncommon Pathogen, *Lactobacillus*, Causing Infective Endocarditis: Case Report and Review

**DOI:** 10.1155/2020/8833948

**Published:** 2020-11-05

**Authors:** Muhannad Antoun, Yousef Hattab, Fadi-Al Akhrass, Leigh Danielle Hamilton

**Affiliations:** ^1^Infectious Disease Department, Pikeville Medical Center, Pikeville, KY, USA; ^2^Pulmonary and Critical Care Department, Pikeville Medical Center, Pikeville, KY, USA; ^3^Clinical Pharmacy Manager, Pikeville Medical Center, Pikeville, KY, USA

## Abstract

*Lactobacillus* is not a common pathogen; however, it can contribute to opportunistic infections such as infective endocarditis (IE). Nonetheless, it has been reported as case reports in correlation with increased probiotic use, dental caries, and intravenous drug abuse.

## 1. Introduction

*Lactobacillus* is Gram-positive rod bacteria, which is typically part of normal flora that exists in oral cavity and gastrointestinal tract. However, it can be pathogenic once it is isolated from sterile sites, such as bloodstream, spinal fluid, and endocardium tissue. Many risk factors have been reported and correlated with infections including decompensated liver cirrhosis, excessive ingestion of probiotic, poor dental hygiene, and others.

## 2. Case Report

The patient in this case was a 40-year-old man with a history of uncontrolled diabetes mellitus and a hemoglobin A1c of 10.7. The patient had a history of uncontrolled blood sugar and diabetic ketoacidosis. The patient also had a history of using illicit drugs in previous years. Nevertheless, upon admission, he was found to be on daily sublingual buprenorphine. The patient was also a smoker, averaging one pack of cigarettes per day. Finally, it was noted that the patient had very poor dentation, and multiple caries were noted upon physical oral exam.

The patient was initially brought to hospital by Emergency Medical Services (EMS).

The patient's family found him to be lethargic with a distinct fruity smell from his mouth. Two weeks prior to admission, the patient was complaining of multiple symptoms, including increased leg swelling, shortness of breath, fatigue, nausea, vomiting, and poor appetite. There was no report of fever, chills, chest pain, or cough. He had history of penicillin allergy causing skin rash. The patient's history was obtained from the patient's mother.

The patient's vital signs on admission were temperature, 98.5 Fahrenheit (F); blood pressure, 98/64 millimeter of mercury (mm Hg); pulse, 101 beats per minute; and respiratory rate, 24 per minute. Cardiac exam showed tachycardia with III/VI systolic murmur. Lungs auscultation showed crackles at the bases, but no wheezing. Lower extremities had +2 pitting edema. Oral cavity showed very poor dentition, multiple caries, and necrotic teeth.

Other patient labs showed elevated blood sugar, up to 1000 milligrams per deciliter (mg/dl); white blood cell count, 29 thousand per cubic milliliter (K/*µ*L); creatinine, 2.20 milligrams per deciliter (mg/dl); ALT, 15 units per liter (U/L); sodium, 141 millimoles per liter (mmol/L); and procalcitonin, 3.59 nanograms per milliliter (ng/mL). Troponin was less than 0.015 ng/mL. Blood gases showed pH, 6.9; lactic acid, 5.3 mmol/L; and blood glucose, 1060 mg/dl; urine drug screen was negative, and urine analysis showed no leukocytes.

A computed tomography (CT) of brain was performed and was negative for any acute finding.

**Figure 1:**
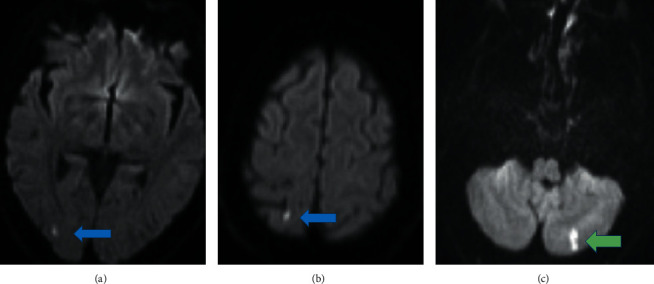
MRI of brain without contrast. Blue arrows: occipital and parietal tiny infarcts. Green arrow: cerebellar infarct.

However, magnetic resonance imaging (MRI) of brain without contrast showed small acute cortical infarct in the left cerebellar hemisphere with two another tiny acute embolic infarcts in the right parietal and occipital lobes (Figure 1).

**Figure 2:**
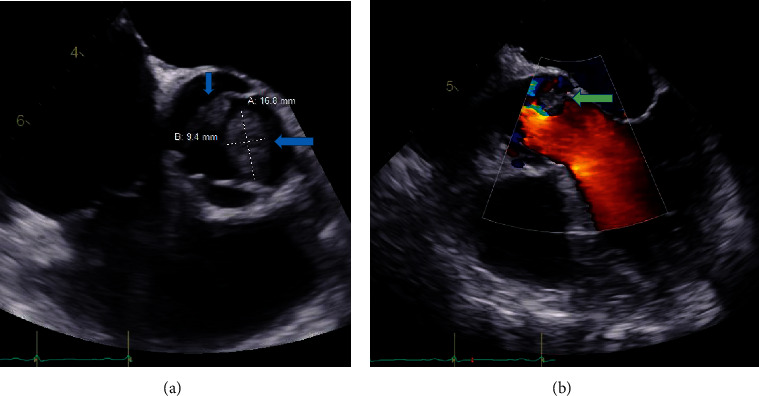
(a) TEE image showed large vegetation (large blue arrow) and another small vegetation (small blue arrow). (b) TEE color Doppler showed AV regurgitation and the vegetation over the valve leaflet (green arrow).

Chest X-ray showed bilateral lung congestion. Two sets of blood culture were obtained and sent to microbiology laboratory. He was started on broad-spectrum intravenous (IV) antibiotic vancomycin and cefepime. Two-dimensional transthoracic echocardiogram study was technically difficult and suboptimal in quality; thus, transesophageal echocardiogram (TEE) was performed, and it showed multiple echodensities compatible with vegetations, associated with moderate aortic valve insufficiency, and mild aortic stenosis. The largest vegetation measures 1.6 × 0.9 cm (Figures 2(a) and 2(b)).The estimated left ventricular ejection fraction was approximately 30%.

Subsequently, the patient's blood cultures turned positive for Gram-positive rods, which were identified as *Lactobacillus rhamnosus*. Susceptibility tests were requested from microbiology laboratory, and it was manually performed using the broth dilution minimum inhibitory concentration (MIC) method. The results showed sensitivity to ampicillin with MIC equal to 1 microgram per milliliter (mg/ml), clindamycin with MIC equal to or less than 0.06 (mg/ml), erythromycin with MIC equal to or less than 0.06 (mg/ml), with penicillin MIC equal to 0.5 (mg/ml) and resistant to vancomycin with MIC higher than 32 (mg/ml). Due to the patient's history of penicillin allergy, the antibiotics were adjusted to IV meropenem. After 96 hours from admission, repeated blood culture became sterilized. Oral surgery was consulted, and the patient underwent full teeth extraction. Nevertheless, the identified source of the bacteremia was from the oral cavity. Due to the size of the vegetation and signs of congestive heart failure, the patient was evaluated by cardiothoracic surgeon and underwent surgery with aortic valve replacement using 23 mm Mosaic porcine tissue valve. Notes from surgery showed a large vegetation on the fused right and left coronary cusp, almost filling the aortic valve and also another vegetation on the noncoronary cusp. Tissue valve culture results showed the same organism isolated from the blood upon arrival to the hospital. Pathology of the excided aortic valve reported myxoid degeneration, focal acute inflammation with fibrinous exudate containing bacterial colonies.

The patient clinically responded well to antibiotic treatment and was discharged home to complete six weeks of IV meropenem. A follow-up and two-dimensional echocardiogram was scheduled after 3 months of end therapy, and it showed improvement in the ejection fraction up to 70%, and the bioprosthetic aortic valve was functioning normally with no signs of regurgitation.

## 3. Discussion

*Lactobacilli* are Gram-positive rods and facultative anaerobic bacteria. Most common are *L. rhamnosus*, *L.* casei, *L. fermentum*, *L. gasseri*, *L. plantarum*, *L. acidophilus*, and *L. ultunensis*. These organisms are normally found in the oral cavity, gastrointestinal tract, and genitourinary tract as normal flora. They can cause invasive infections such as bacteremia, endocarditis, peritonitis, and meningitis.

*Lactobacillus* endocarditis was first reported in 1938 by Dr. Marchall F [1].

There are many sources of exposure to *lactobacilli*. These sources include probiotics, fermented foodstuffs (e.g., yogurt, cheese, sauerkraut, and other fermented vegetables). In healthy humans, *lactobacilli* are normally present in the oral cavity (103–104 colony-forming units per gram (cfu/g), the ileum (103–107 cfu/g), and the colon (104–108 cfu/g), and they are the dominant microorganism in the vagina [2].

Usually immunocompromised patients are more susceptible to opportunistic infections from these bacteria. However, there is no evidence that consumption of probiotics increases the risk of opportunistic infection among this group of patients. No increases in infection were noticed in HIV-infected patients consuming probiotics [3], which supports the safety of probiotics in such a group.

Cases of infection due to *lactobacilli* and *bifidobacteria* are very rare and are estimated to represent 0.05%–0.4% of cases of infective endocarditis and bacteremia [4].

Griffith et al reviewed 39 *Lactobacillus* endocarditis cases reported in the literature. The response to medical therapy alone was low (39%), and mortality rate was (27%). The possible reasons were unreliable antimicrobial susceptibility studies and lack of standardized therapy [5]. One case infected with *Lactobacillus acidophilus* was cured by medical therapy alone. A combination synergistic therapy with penicillin and aminoglycoside was effective and optimal therapy.

However, another case infected with *Lactobacillus casei* subspecies *rhamnosus* required surgical replacement to the infected valve. This organism was resistant to many antibiotics.

Underlying diseases are an important factor in developing invasive *Lactobacillus* infection. Based on the review of 45 patients with *Lactobacillus* bacteremia between 1979 and 1994 by Husni R.N and Gordon S.M at the Cleveland Clinic Foundation, it showed multiple predisposing factors including cancer (40%), recent surgery (38%), and diabetes mellitus (27%). 11 of those patients were receiving immunosuppressive therapy, 11 were receiving total parenteral nutrition, and 23 had received antibiotics without activity against *Lactobacillus* [6].

A retrospective review of 200 *Lactobacillus*-associated infection cases, which was published in *European Journal of Clinical Microbiology* in 2005 by Cannon et. al, revealed cases with endocarditis, peritonitis, and meningitis. The most common isolated species were *L*. *casei* and *L*. *rhamnosus*. It was sensitive to erythromycin and clindamycin and resistant to vancomycin. The mortality rate was 30% which was related to inadequate treatment (*P*=0.001) and polymicrobial bacteremia (*P*=0.044). Of these cases, 73 patients had endocarditis, and the majority of these patients had underlying structural heart disease (63%) or dental condition (47%) [7].

In 1999, Mackay et al. reported mitral valve endocarditis (MV-IE) due to *L. rhamnosus* in a patient with a history of MV prolapse, after self-medication with freeze-dried probiotic preparation. The patient was treated medically with synergistic gentamicin and ampicillin [8].

Another case by Presterl E. reported an aortic valve (AV) endocarditis due to *L. rhamnosus*, associated with excessive yogurt ingestion. That patient was treated medically followed by aortic valve replacement [9].

A probiotic-related *Lactobacillus rhamnosus* AV and MV endocarditis was reported in a young woman with alcoholic liver cirrhosis (Child's Pugh Class B). The patient had recurrent *Clostridium difficile*-associated colitis; thus, she was self-medicating with daily use of a commercially available probiotic formulation (containing *Lactobacillus acidophilus* (32 billion CFU organisms), *Lactobacillus rhamnosus* (4 billion CFU organisms), and *Saccharomyces cerevisiae* (4 billion CFU) for 7 months. The patient died despite medical and surgical therapies [10].

Our patient was at risk for infection due to uncontrolled diabetes mellitus and poor dentition. We believe oral hygiene is a probable risk factor for invasive *Lactobacillus* infection and endocarditis. Treatment options for this type of infection follows the same treatment guidelines as for endocarditis including medical and surgical interventions therapies.
